# The anatomy of AI implementation skepticism in Polish healthcare: an explanatory mixed-methods analysis of psychographic barriers among healthcare professionals

**DOI:** 10.3389/fpubh.2026.1866364

**Published:** 2026-06-15

**Authors:** Jarosław Garski, Dariusz Walkowiak

**Affiliations:** Department of Organization and Management in Health Care, Poznan University of Medical Sciences, Poznań, Poland

**Keywords:** digital health literacy, digital public health, health innovation, healthcare AI, healthcare management, healthcare professionals, implementation science, technology resistance

## Abstract

Healthcare organizations struggle to scale AI, often attributing failures to a skills gap in the workforce. However, this mixed-methods study of Polish healthcare professionals reveals that professional roles, whether physician or executive, do not predict AI resistance well. Instead, resistance clusters into four psychographic profiles: Skeptics, Cost-Focused, Pragmatists, and Optimists. As its primary novel contribution, this research reveals that what is commonly captured in survey instruments as a “lack of expertise” actually masks distinct organizational defense mechanisms, including institutional exhaustion, fears of legal liability, and operational budget constraints. By demonstrating that stated competency gaps are proxies for systemic distrust rather than simple educational deficits, this study moves beyond traditional barrier counting to challenge demographic-based technology implementation. For healthcare leaders and researchers, the implication is clear: successful AI adoption requires abandoning generic, role-based training in favor of targeted change management that addresses the underlying psychological and structural barriers driving technological resistance.

## Background

The integration of artificial intelligence into healthcare delivery systems has been characterized as one of the most consequential technological transitions of the 21st century (1, 2). Applications ranging from diagnostic imaging analysis to clinical decision support, predictive analytics, and natural language processing for medical documentation have demonstrated measurable improvements in diagnostic accuracy, operational efficiency, and patient outcomes in controlled settings (3). Yet the translation of these demonstrated capabilities into routine clinical practice remains markedly slow. Global survey data consistently indicate that fewer than 15% of healthcare organizations have moved beyond pilot-phase AI deployments (4), a trend that persists despite favorable regulatory signals from the European Union’s AI Act framework (5) and accelerating vendor activity in health technology markets.

Implementation science offers robust theoretical frameworks for analyzing multi-level barriers to adoption. The Consolidated Framework for Implementation Research (CFIR), originally proposed by Damschroder et al. (6) and recently updated (7), identifies five interacting domains that determine implementation success: the Innovation itself, the Outer Setting (regulatory environment, funding structures, external pressures), the Inner Setting (organizational culture, available resources, implementation climate), Individuals (knowledge, beliefs, self-efficacy of those involved), and the Implementation Process. CFIR has increasingly been applied to research on healthcare AI adoption (8–10), providing a multi-level lens that captures the systemic nature of implementation barriers.

The CFIR framework suggests that barriers to healthcare AI will be distributed across multiple system levels, a prediction directly testable through quantitative barrier mapping and qualitative exploration of underlying mechanisms.

Despite the availability of these frameworks, most empirical studies of AI barriers in healthcare have relied on simple frequency counts of reported obstacles (1, 11), without examining whether barrier profiles differ systematically across professional subgroups or, critically, whether stated barriers reflect genuine operational constraints or mask deeper psycho-organizational dynamics. The five barrier dimensions assessed in this study expertise, infrastructure, legal/regulatory, organizational culture, and costs map directly onto CFIR domains: expertise maps to the Individuals domain (Knowledge and Beliefs, Self-Efficacy); legal/regulatory challenges map to Outer Setting (Policies and Laws); costs span Outer Setting and Inner Setting (External Funding, Available Resources); technical infrastructure maps to Inner Setting and Innovation (Available Resources, Complexity); and organizational culture maps to Inner Setting (Implementation Climate, Culture). This domain mapping provides a theoretically grounded structure for both quantitative assessment and qualitative investigation.

The Polish healthcare system provides a particularly informative context for examining these dynamics. As a post-socialist system undergoing simultaneous digital transformation and structural reform, it combines the professional hierarchies characteristic of Western European medicine with the institutional legacies of centralized planning: chronic underfunding, bureaucratic inertia, and workforce aging (12, 13). Poland’s healthcare workforce is among the oldest in the EU, with the median age of physicians exceeding 50 years. The system operates under a single-payer model (National Health Fund, Narodowy Fundusz Zdrowia) while accommodating a growing private sector that now accounts for approximately 30% of healthcare service delivery (14). Prior digitalization efforts, including the mandatory transition to electronic medical documentation (EDM), have generated mixed results, with many professionals reporting that digitalization increased rather than decreased administrative burden (13).

Despite these contextual factors, existing research on AI readiness in Polish healthcare has relied predominantly on single-method quantitative surveys with role-based analytical frameworks (15). No published study has combined population-level barrier measurement with qualitative investigation of the mechanisms underlying those barriers in the Polish context.

While the empirical material in this study is drawn from the Polish healthcare context, the implementation problem it addresses is not country-specific. Comparative analyses of AI maturity across OECD healthcare systems show that even high-resource jurisdictions struggle to translate strategic intent into operational adoption, with most countries lacking unified governance mechanisms for AI integration (16). A further structural gap, the “AI digital divide”, has been documented in financially, racially, and geographically disadvantaged health-care settings, where reduced AI access compounds pre-existing inequalities (17). The mechanisms surfaced in this study – institutional exhaustion, liability anchoring, and budget-constrained pragmatism – are plausibly generalizable to any healthcare system in which professionals operate under chronic resource pressure and evolving regulatory regimes. The psychographic segmentation method demonstrated here is offered as a transferable analytical instrument, not a country-bound finding.

### Mixed-methods justification

Quantitative surveys can identify barriers at the population scale and assess their relative magnitude, but they cannot explain why these barriers persist or how they operate within the socio-technical reality of clinical workflows. Qualitative in-depth interviews are necessary to access the experiential accounts, organizational narratives, and psychological defense mechanisms that statistical distributions cannot capture. This study, therefore, employs an Explanatory Sequential Mixed-Methods Design (18), in which a dominant quantitative phase is followed by a supplementary qualitative phase whose explicit purpose is to explain, elaborate, and validate the statistical findings. This design has been recommended for healthcare implementation research precisely because it allows researchers to identify unexpected statistical patterns (such as null findings or paradoxical correlations) and then seek explanatory mechanisms through purposive qualitative inquiry (19).

This study addresses three research questions:

- *RQ1* (Quantitative): Do professional role, sector type, or self-reported AI knowledge predict barrier perception among Polish healthcare professionals?
- *RQ2* (Qualitative): What organizational and psychological mechanisms explain the barriers identified in the quantitative phase?
- *RQ3* (Mixed-Methods): How do qualitative narratives validate and extend the psychographic cluster model derived from quantitative analysis?

## Methods

### Study design

This study employed an Explanatory Sequential Mixed-Methods Design (18), in which a dominant quantitative phase preceded a supplementary qualitative phase whose explicit purpose was to explain, elaborate, and validate the statistical findings. The quantitative strand held methodological priority (QUAN qual), and the temporal sequence was strictly maintained: qualitative data collection commenced only after completion of quantitative analysis. This design aligns with the Good Reporting of a Mixed Methods Study (GRAMMS) guidelines (20) and the APA Journal Article Reporting Standards for Mixed Methods (JARS-Mixed).

### Phase 1 - quantitative strand

#### Data collection

A cross-sectional online survey was conducted between April and September 2025 using Google Forms. The target population comprised healthcare professionals employed in Polish healthcare facilities, including managers and administrative staff, physicians, and allied medical professionals. A combination of purposive and snowball sampling was employed to reach respondents across public, private, and non-clinical healthcare entities. The non-probability sampling strategy was selected due to the absence of a comprehensive national registry of healthcare professionals stratified by role category and the need to oversample managerial roles for adequate subgroup analysis. A total of 330 complete responses were obtained.

#### Instrument

The survey instrument comprised 39 items across six domains (21), operationalizing five barrier dimensions: (1) Lack of expertise and skilled personnel, (2) Technical infrastructure and data security, (3) Legal, ethical, and regulatory challenges, (4) Organizational culture and workforce resistance, and (5) High implementation and maintenance costs. Items were measured on 5-point Likert scales (1 = strongly disagree to 5 = strongly agree). Internal consistency was assessed using Cronbach’s alpha, yielding a composite reliability of *α* = 0.844, exceeding the conventional threshold of 0.70 (22–24). The overall study design follows established principles for mixed-methods research in the social and health sciences (18).

The 39-item instrument was developed through a structured process. First, an item pool was generated from a review of published AI-implementation barrier inventories in healthcare and adjacent sectors, mapped to the five CFIR-aligned barrier domains (lack of expertise, legal/ethical challenges, technical infrastructure, organizational culture, implementation costs). Second, items were reviewed for face and content validity prior to deployment, and items judged ambiguous or redundant were revised or removed. Third, internal consistency reliability across the final 39-item scale was Cronbach *α* = 0.844, indicating acceptable reliability for the multi-dimensional barrier construct. The five barrier dimensions used in the clustering analysis were defined *a priori* from the CFIR framework rather than derived *post hoc* through exploratory factor analysis; this design choice preserves the theoretical mapping of dimensions to recognized implementation constructs.

While Cronbach *α* confirms acceptable internal consistency of the full 39-item scale, the present design relies on *a priori* CFIR-grounded construct definition rather than post-hoc factor extraction. Confirmatory factor analysis on an independent sample is a productive next step for instrument validation; conducting EFA on the same data subsequently used for cluster derivation would introduce circular validation concerns and was therefore not performed here.

#### Statistical analysis

Statistical analysis comprised descriptive statistics; Kruskal–Wallis *H* tests with Conover-Iman *post hoc* comparisons (Bonferroni correction) for three-group professional role comparisons; Mann–Whitney *U* tests for two-group sector comparisons (public vs. private); and Spearman rank-order correlations to assess the association between self-reported AI knowledge and barrier perception. K-means cluster analysis was then performed on the five composite barrier dimensions (Lack of expertise, Technical infrastructure & data security, Legal/ethical/regulatory challenges, Organizational culture, and Implementation costs) to identify distinct respondent segments based on their barrier-perception profiles. The elbow method indicated an optimal solution of K = 4 clusters. Cross-tabulation with Chi-square testing and adjusted residuals was used to determine the statistical over-indexing of specific professional roles within the identified clusters. Readiness was classified using Likert-based thresholds: Low (≤2.5), Medium (2.6–3.5), and High (≥3.6) (25, 26).

### Phase 2 – qualitative strand

#### Participant selection

Thirteen senior healthcare managers were purposively selected for semi-structured in-depth interviews (IDIs). Participant selection was informed by the quantitative results to ensure representation across the power hierarchy identified in Phase 1, constituting the “connecting” integration point. The sample comprised five general/strategic managers, four medical division directors, and four operational/financial directors, representing both large city hospitals and small county hospitals. Respondents were identified by functional codes [Role_Category-Facility_Type] per an anonymization matrix.

The qualitative informant pool was purposely limited to senior healthcare managers (directors, deputy directors, heads of clinical departments managing administrative responsibilities, and representatives of national managerial associations) for both operational and methodological reasons. From an operational perspective, this group represents the primary decision-makers tasked with budgeting, initiating, and overseeing AI implementation within their facilities, many of whom have already attempted or are currently navigating these deployments in practice. Methodologically, the quantitative phase identified that managers constitute the only professional group whose barrier perception diverges significantly from other roles, specifically on the Implementation Costs dimension (Mann–Whitney *U* = 10,700.5, *p* = 0.002). Under the Explanatory Sequential design (18), the qualitative phase is positioned to explain the statistically observable patterns identified in Phase 1. Consequently, broadening the informant pool to frontline clinicians, nurses, or IT staff—professional groups whose barrier profiles did not diverge significantly in Phase 1—would not generate additional explanatory leverage for the specific statistical pattern under investigation, nor would it capture the strategic insights unique to those actually driving the technological adoption.

#### Interview protocol

A semi-structured interview guide was organized around four thematic blocks, developed after completion of quantitative analysis to probe specific statistical findings: (1) the competency consensus phenomenon, (2) cost divergence between professional groups, (3) the public-private sector equivalence, and (4) the knowledge-barrier paradox. Each interview concluded with an open-ended question asking participants to identify a single systemic change required for AI implementation.

#### Analysis

Thematic analysis was conducted following the Braun and Clarke framework (27, 28). Findings were classified by frequency: dominant pattern (≥7 independent sources) and marginal observation (<7 sources). Thematic coding was conducted manually with systematic cross-case comparison across all 13 transcripts.

#### Sample size justification

Guest et al. (29) demonstrated that thematic saturation in purposive samples is typically reached within 12 interviews. The present sample of *N* = 13 exceeds this threshold, and systematic monitoring of theme recurrence across the final three interviews confirmed that no new thematic categories emerged.

#### Mixed-methods integration strategy

Integration occurred at two methodological junctures. First, at the design level, connections were made: quantitative results directly informed IDI participant selection criteria and the development of the interview protocol. Second, merging was achieved at the interpretation level: quantitative cluster profiles were merged with IDI thematic summaries through narrative weaving in the Results and Discussion sections and through a Statistics-by-Themes Joint Display (Table 1) (18, 30). This dual integration strategy ensures that the qualitative phase functions as an explanatory mechanism for statistical findings rather than as a parallel, disconnected analysis.

**Table 1:** Statistics-by-themes joint display.

Psychographic persona (cluster)	Quantitative profile	Dominant IDI theme	Representative excerpt [role-facility]	Meta-inference
Skeptics (*N* = 90, 27.3%)	Four of five barriers are highest among all clusters; all barriers >4.2; over-indexed in managers (+7%).	Resistance functions as a defense mechanism rooted in systemic exhaustion and demographic-psychological factors, rather than in a rational assessment of specific barriers. Prior digitalization cycles generated bureaucratic burden, creating institutional distrust toward all technology promises.	*“Few believe that this can actually help streamline their work or reduce their burden. This is an alien creature that excites some, but the system’s unpredictability - another health fund reform, another restructuring means that experienced managers fear investing in anything”* [General_Management-National Association]	Undifferentiated resistance is primarily psychological in origin, systemic exhaustion and status quo protection, not technical illiteracy. Trust-building through pilot demonstrations must precede any technical deployment for this group.
Cost-focused (*N* = 72, 21.8%)	Highest cost (4.27) among all clusters, and high tech/security (4.20); moderate culture (3.30); dominated by managers (58%).	Cost concern reflects immediate operational reality, payroll, IT licensing, and infrastructure, not philosophical opposition. Budget constraints serve as both genuine barriers and rhetorical shields for deeper uncertainty about ROI.	*“Every innovative implementation is very costly and requires external funding sources, which take years to secure. In a public hospital, financial return is not even how we think we invest to maintain quality, not to generate profit”* [Operational_Financial-Large_City_Hospital]	Financial barriers reflect immediate operational reality, not philosophical opposition. Cultural adoption is contingent on prior infrastructure investment. This group is persuadable with a robust, sector-specific ROI case.
Pragmatists (*N* = 98, 29.7%)	High expertise (4.17) and culture (3.80); moderate cost (2.83); over-indexed in physicians (+10%).	Clinical liability anchoring: physicians and medically trained managers fear legal consequences of algorithm override criteria and liability frameworks.	*“Everything is fine until I have to take responsibility for that description. If AI generates it, a human will still stand behind it and bear the legal consequences. The technology is not yet refined enough to use without significant reservations”* [Operational_Financial-Small_County_Hospital]	Clinician resistance is rational and liability-anchored. Targeted protocol training that addresses professional autonomous judgment is required; generic AI awareness campaigns provide no benefit to this group.
Optimists (*N* = 70, 21.2%)	All barriers generally low to moderate <3.1; youngest cohort; over-indexed in other professions (+8%).	Digital-native perspective combined with frustration at legacy analog infrastructure. Perceive existing organizational inertia and generational resistance as the real barriers, not the technology itself.	*“I dream of a system where the doctor can finally be a doctor, and everything is recorded automatically. The younger generation immediately catches everything they are open to innovation and understand how to use it”* [Medical-Small_County_Hospital]	This cohort is an underutilized change asset. Peer mentoring and internal champion roles can leverage their low perceived resistance as a grassroots diffusion mechanism within resistant organizational cultures.

#### Ethical considerations

This study adhered to the Declaration of Helsinki and received a formal waiver from the Bioethics Committee at the Poznan University of Medical Sciences (Ref: KB-268/25). As the project was classified as a survey study rather than a medical experiment under Polish law and Good Clinical Practice (GCP) standards, formal ethical approval was not required. All participants provided voluntary, informed consent via the web-based questionnaire prior to data collection. To ensure anonymity, In-Depth Interview (IDI) participants were assigned functional codes; no identifiable patient data were collected.

## Results

### Sample characteristics

The quantitative sample (*N* = 330) was predominantly female (64.2%), with a median age category of 51–60 years and extensive professional experience (47.6% reported >20 years). Professional role distribution comprised managers and administrative staff (*n* = 156, 47.3%), physicians (*n* = 77, 23.3%), and allied medical professionals (*n* = 97, 29.4%). Sector distribution reflected the structure of the Polish healthcare system: public facilities (*n* = 195, 59.1%), private facilities (*n* = 101, 30.6%), and non-clinical healthcare entities (*n* = 34, 10.3%). Self-reported Al knowledge was moderate (*M* = 2.98 on a 5-point scale). Nearly half (45.5%) reported currently using AI tools, while 43.9% expressed openness to future use; only 7.9% reported no plans to engage with AI. A complete descriptive breakdown of the sample by professional role, facility type, ownership sector, and years of professional experience is provided in Supplementary Table S1.

The qualitative sample (*N* = 13) comprised senior healthcare managers representing five general/strategic directors, four medical division directors, and four operational/financial directors across large city hospitals (n = 7) and small county hospitals (*n* = 6).

### Barrier hierarchy: the competency consensus

The statistical prominence of the expertise barrier was directly confirmed in the qualitative phase (see Table 2). Thematic analysis across the IDIs identified a dominant pattern (present in ≥10 of 13 interviews): respondents consistently described AI as “something fundamentally different from previous IT systems” and framed the expertise deficit not as a simple technical skills gap, but as a compound construct encompassing fear of job displacement, anxiety about legal liability for algorithmic errors, and deep-seated distrust rooted in negative experiences with prior digitalization mandates. As one respondent articulated:

**`TABLE 2:** Hierarchy of perceived AI implementation barriers (*N* = 330).

Rank	Barrier dimension	Mean	SD	Interpretation
1	Lack of expertise and skilled personnel	4.00	0.82	Critical consensus
2	Technical infrastructure and data security	3.86	0.77	High priority
3	Legal, ethical, and regulatory challenges	3.85	0.85	High priority
4	Organizational culture and workforce resistance	3.64	0.89	Moderate concern
5	High implementation and maintenance costs	3.56	1.01	Lowest priority/highest variance

*“This is not a lack of competence; it is fear of the unknown. It is a defense mechanism. People are afraid for their jobs, and when you look at the age groups, the vast majority are people aged fifty and above who are less inclined toward innovation by nature.” [Operational_Financial-Large_City_Hospital]*

This finding supports the interpretation of the competency barrier as a psychological construct rather than a purely technical one. The qualitative data reframes “lack of expertise” as an umbrella term that masks psycho-organizational resistance, a distinction with direct implications for intervention design.

### Professional role comparisons

Kruskal-Wallis tests revealed statistical convergence across professional groups in four of five barrier dimensions (Table 3).

**Table 3:** Kruskal–Wallis test results: barrier perception by professional role.

Barrier dimension	*H*	*p*	*η*^2^	df	Interpretation
Lack of expertise	2.831	0.243	0.003	2	Not significant
Legal/ethical challenges	3.661	0.160	0.005	2	Not significant
Technical infrastructure	5.935	0.051	0.012	2	Not significant (borderline trend)
Organizational culture	0.486	0.784	0.000	2	Not significant
Implementation costs	12.462	0.002	0.032	2	Significant (small effect)

While technical infrastructure showed a borderline trend toward significance (*p* = 0.051), the only statistically robust divergence appeared in the financial domain, where managers (Med = 4.0, IQR = 3.0–5.0) rated cost barriers significantly higher than physicians (Med = 3.5, IQR = 2.5–4.0). Median and IQR values for each barrier dimension, stratified by professional role, are reported in Supplementary Table S2. The qualitative phase provided a contextual explanation for both the consensus and this single point of divergence. Thematic analysis of the managerial divide (Block 2 of the interview protocol, which addressed perceived barriers and resistance mechanisms; see Methods) revealed a dominant pattern: among managers, cost concerns reflected immediate operational pressures, payroll obligations, infrastructure maintenance, and IT system licensing rather than philosophical opposition to technology. As one financial director explained:

*“Time is money, and we begin with money and end with money. We already pay approximately 400,000 PLN annually just to maintain existing IT programs. That is not a small sum for a hospital like ours.” [Operational_Financial-Small_County_Hospital]*

The cost difference between managers and physicians, while statistically significant, was reinterpreted by the qualitative data as reflecting differential proximity to fiscal operations rather than fundamentally different attitudes toward AI. Physicians perceived costs abstractly, while managers confronted them as daily operational constraints. Neither group was philosophically opposed to AI; they operated from different information environments regarding institutional finances.

### Sector analysis

Mann–Whitney U tests revealed no statistically significant differences between public and private sector respondents across any barrier dimension [all *p* > 0.05: costs (*U* = 10,700.5, *p* = 0.216), expertise (*U* = 9,434.5, *p* = 0.550), legal/ethical (*U* = 9,509.5, *p* = 0.628), technical infrastructure (*U* = 9,515.0, *p* = 0.633), and organizational culture (*U* = 10,127.5, *p* = 0.688)].

Qualitative data provided a direct explanatory mechanism for this unexpected null result. A dominant pattern across the IDIs (present in ≥9 of 13 interviews) identified workforce homogenization as the primary causal factor. Respondents consistently noted that medical and managerial personnel circulate between public and private institutions, carrying identical competency deficits, resistance patterns, and institutional anxieties across sectoral boundaries:

*“It does not matter what form of ownership we are discussing. It depends on the people. And given the migration of all kinds of medical and managerial staff between public and private entities, these are the same people.” [General_Management-Clinical_Hospital]*

Additionally, a marginal observation (5 of 13 sources) identified a paradoxical mechanism: private-sector short-term profit orientation actually increases risk aversion toward experimental technologies with unclear return on investment. Several respondents noted that private entities “watch every penny more” and are paradoxically less willing than public hospitals to absorb the financial risk of unproven AI implementations.

### The knowledge paradox

Spearman correlation analysis revealed no significant association between self-reported AI knowledge and barrier perception across any dimension. The strongest coefficient was observed for expertise barriers (*ρ* = −0.079, *p* = 0.153), with all other correlations hovering near zero (legal: *ρ* = −0.049, *p* = 0.373; technical: *ρ* = 0.005, *p* = 0.929; culture: *ρ* = 0.031, *p* = 0.573; excluding the costs dimension, which is addressed in the role comparison section above). The qualitative phase offered a reframing of this null result. A dominant pattern across the IDIs identified two complementary mechanisms. First, genuine technical familiarity does not significantly decrease perceived barriers as one might expect, but rather transforms them; it reveals the probabilistic limitations, error rates, and liability implications of algorithmic systems that laypeople do not perceive, thereby tempering the expected reduction in barrier perception:

*“The more we know, the more critically we approach AI. The less we know, the less critically we approach it. People with high knowledge feel its risks and threats - and that is actually an advantage, because they protect themselves and others.” [General_Management-Large_City_Hospital]*

Second, a marginal observation (6 of 13 sources) identified inflated self-assessment: many respondents who declared “high knowledge” of AI operated at a popular-science level of understanding, using colloquial framing that revealed superficial engagement with the technology. As one medical director observed:

*“Doctors always rate themselves very highly. But if you go into specifics, their knowledge is at the level of popular-science articles.” [Operational_Financial-Large_City_Hospital]*

This finding has direct implications for survey-based knowledge measurement: the null correlation partly reflects a measurement artifact rather than a genuine absence of a relationship.

### Psychographic segmentation

The four-cluster solution was selected through a two-stage validation procedure: statistical optimization via the elbow method, followed by independent qualitative validation against the IDI thematic patterns. K-means clustering applied to the five composite barrier dimension means identified four distinct psychographic personas (Table 4).

**Table 4:** Psychographic cluster profiles (K = 4).

Persona	*N* (%)	Costs (outer/inner)	Culture (inner)	Expertise (individuals)	Tech/security (inner/innovation)	Legal/ethical (outer)	Role composition
Skeptics	90 (27.3%)	4.22	4.49	4.70	4.58	4.61	Over-indexed in managers (+7%)
Cost-focused	72 (21.8%)	4.27	3.30	3.85	4.20	4.12	Dominated by managers (58%)
Pragmatists	98 (29.7%)	2.83	3.80	4.17	3.65	3.80	Over-indexed in physicians (+10%)
Optimists	70 (21.2%)	3.00	2.65	3.02	2.87	2.90	Over-indexed in other professions (+8%)

Over-indexing derived via adjusted residuals from chi-square cross-tabulation. Barrier centroid values reflect K-means segmentation output.

**Figure 1:**
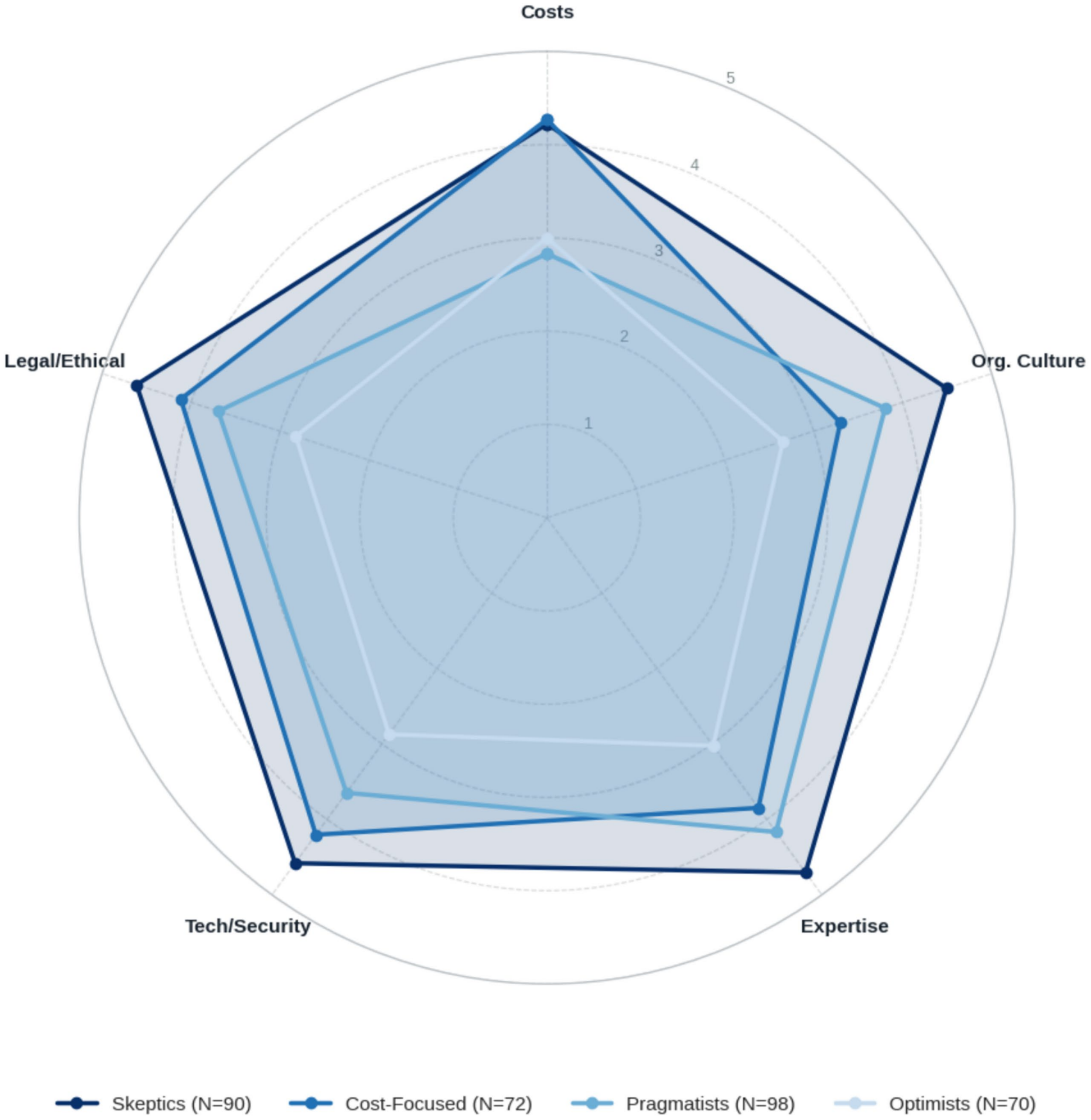
Radar profiles compress five-dimensional Euclidean cluster separation into a two-dimensional polygon representation; visual overlap of polygon outlines does not imply lack of statistical separability in the original five-dimensional feature space. Cluster distinctness was established by the elbow criterion applied to the within-cluster sum of squares (K = 2 through K = 7, inflection at K = 4) and independently corroborated by qualitative thematic mapping against the 13 IDIs (see Methods).

As shown in Figure 1, these overlapping radar profiles illustrate the multidimensional nature of AI resistance. Instead of a monolithic ‘lack of expertise,’ the data reveals four distinct archetypes: Skeptics (uniformly critical), Cost-Focused (budget-anchored), Pragmatists (liability-anchored), and Optimists (inertia-focused). Professional role membership did not determine cluster assignment: all four clusters contained representatives from all three professional groups, confirming that psychographic profiles cross-cut demographic categories.

### Joint display: statistics-by-themes integration

This is the centerpiece of the integration in the mixed-methods design. Table 1 presents the systematic merging of quantitative cluster profiles with qualitative thematic findings.

## Discussion

### Professional role as a weak predictor

This subsection examines why the quantitative analysis failed to detect role-based differences in barrier perception, and what this null finding implies for implementation theory.

The quantitative findings of this study provide strong evidence that professional role is a weak predictor of the AI barrier perception. Four of five barrier dimensions showed no statistically significant differences across managers, physicians, and allied professionals. The single significant divergence - cost perception (*p* = 0.002, *η*^2^ = 0.032) - carried a small effect size that explained only 3.2% of between-group variance. The k-means clustering analysis reinforced this conclusion: all four psychographic clusters contained representatives from all three professional groups, and cluster membership was determined by attitudinal configurations rather than occupational labels.

The qualitative data validated this finding through a convergent pattern: IDI respondents whose profiles strongly aligned with the same cluster exhibited similar linguistic patterns, operational concerns, and emotional framing, regardless of their professional title. A general director and a financial deputy director, whose profiles mapped onto the Cost-Focused cluster, used nearly identical language about daily fiscal pressures, while a medical director and a strategic plenipotentiary, whose narratives aligned with the Pragmatist cluster, shared the same liability-centered framing. This cross-method convergence supports the argument that psychographic profiling offers a superior analytical framework for implementation planning compared to traditional role-based segmentation (31, 32).

These findings align with the CFIR framework’s prediction that implementation barriers are distributed across multiple system levels rather than concentrated within any single professional group (6, 7). The negligible effect sizes for role-based comparisons (*η*^2^ ≤ 0.032) – including the virtually nonexistent effect for the borderline technical infrastructure trend (*η*^2^ = 0.012) – demonstrate that professional role, a variable frequently used to stratify healthcare technology adoption studies, explains virtually no variance in barrier perception when those barriers span CFIR’s Individuals, Inner Setting, and Outer Setting domains simultaneously.

The k-means clustering confirmed that psychographic profiles, cutting across professional categories, provide the analytically superior framework for implementation planning. This is consistent with recent CFIR-based analyses of healthcare AI, in which Finkelstein et al. (8) and Peek et al. (9) found that organizational and individual-level determinants interact in ways that demographic stratification cannot capture. Smith et al. (31) demonstrated that psychographic segmentation in healthcare contexts reveals behavioral patterns invisible to demographic analysis, while Koziel and Shen (32) found that psychographic variables outperformed demographic ones in predicting technology adoption behavior. The role-invariant prominence of the ‘lack of expertise’ barrier identified in the present study is consistent with the psychographic segmentation pattern reported by Smith et al. (31), who similarly found that demographic and role variables explained less variance in technology-resistance attitudes than psychographic profile. The present study extends this literature by providing the first mixed-methods validation of psychographic clusters in the context of a healthcare AI implementation.

### Mechanisms behind the barriers

Building on the quantitative null findings, this subsection draws on the qualitative phase to identify the organizational mechanisms behind the headline barrier rankings.

This subsection constitutes the primary new contribution of the mixed-methods design. Four mechanisms were identified through qualitative analysis that explain patterns invisible to the quantitative data alone. These mechanisms reveal that the barriers captured in the quantitative phase are structured like an iceberg, with visible survey results masking deeper, submerged organizational dynamics.

**Figure 2:**
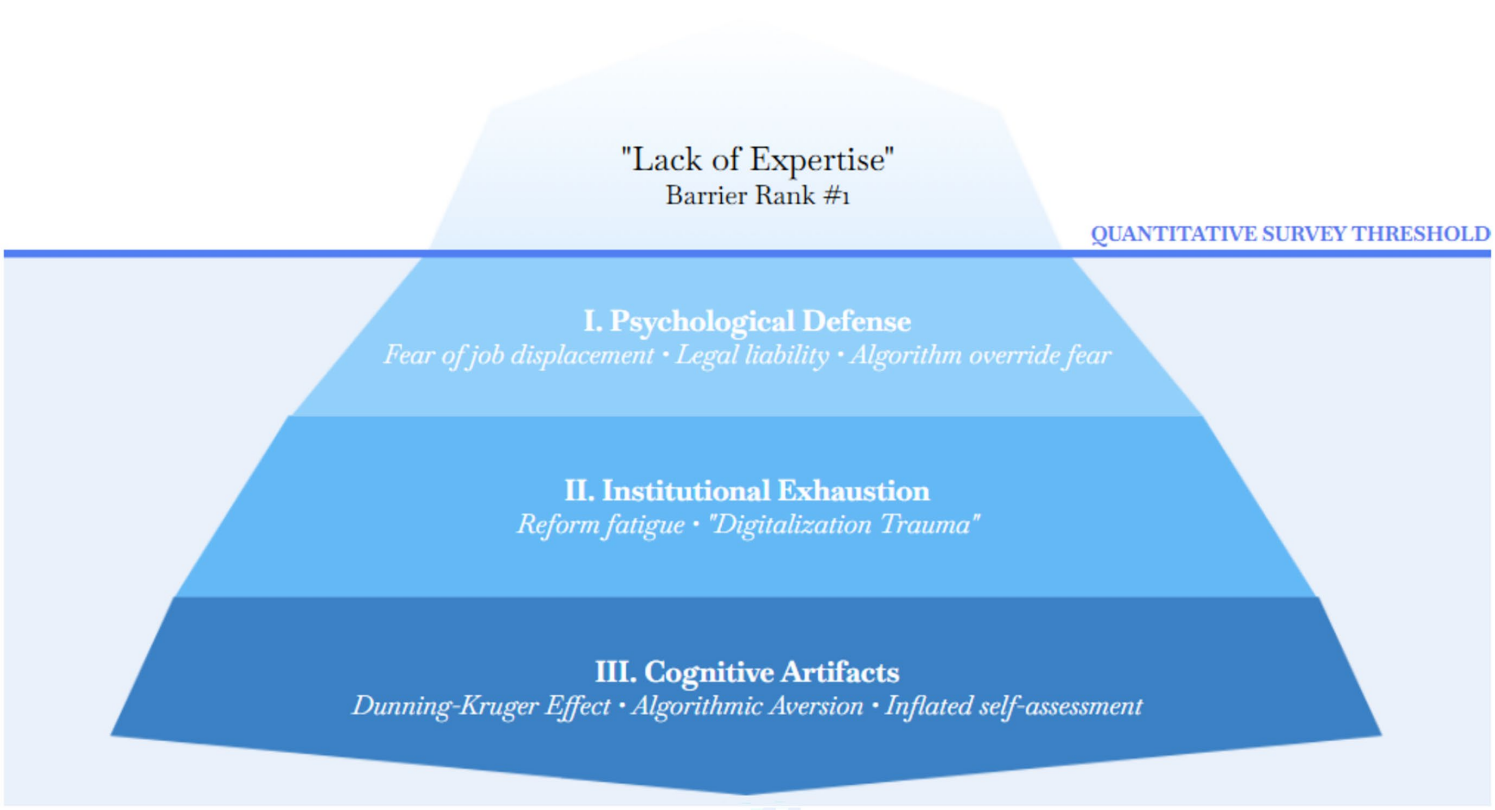
The iceberg model of AI implementation skepticism. The visible tip represents the statistical consensus on “Lack of Expertise” (M = 4.00), while the submerged layers represent the psychological and institutional mechanisms identified during IDI interviews.

These two patterns - the role-invariant prominence of the ‘lack of expertise’ barrier (hereafter the *competency consensus*) and the manager-specific elevation of the cost barrier (hereafter the *cost divergence*) - correspond to the surface and sub-surface levels of the iceberg model presented in Figure 2.

Competency consensus explained. As illustrated in the “tip” of the iceberg (Figure 2), the IDI dominant finding reframes “lack of expertise” as a broad umbrella that combines genuine technical skills gaps with psycho-organizational resistance: fear of job displacement, GDPR liability concerns, and disruption of established workflows converge under a single survey response. Previous digitalization cycles in Polish healthcare, particularly the mandatory transition to electronic medical documentation, generated bureaucratic burden rather than efficiency gains, creating systemic skepticism toward all optimistic technology promises. Multiple respondents described a pattern where new digital mandates increased documentation volume (*from* “10 pages to 42 pages” *in one respondent’s career*) without delivering the promised time savings. This institutional memory shapes current perceptions of AI: the workforce does not evaluate AI on its technical merits but through the lens of prior reform failures. The “lack of competence” response in surveys thus captures a sociological phenomenon, institutional distrust, alongside, rather than merely replacing, a straightforward training gap.

Cost divergence explained. The apparent conflict between managers and physicians over costs dissolves when qualitative context is taken into account.

**Figure 3:**
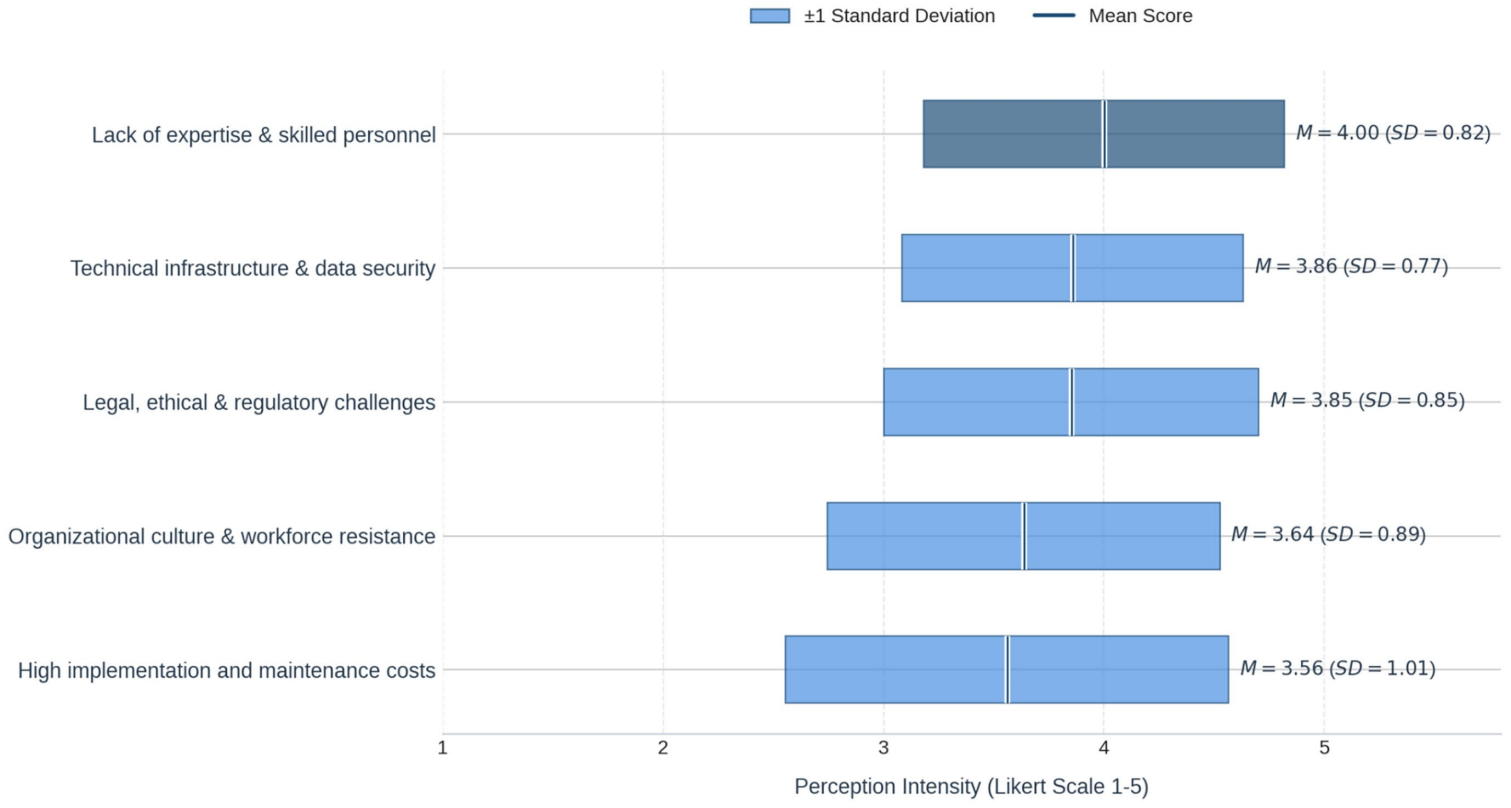
Barrier intensity and variance distribution. Each bar represents one of five barrier dimensions. Mean intensity rating is plotted on a 5-point Likert scale (1 = not a barrier at all; 5 = an absolutely critical barrier); bar width represents ±1 standard deviation across respondents, indicating the degree of inter-respondent agreement on each barrier’s intensity. The “High implementation and maintenance costs” dimension exhibits the highest variance (SD = 1.01), reflecting the polarized financial realities between large clinical centers and resource-constrained county hospitals.

The apparent conflict between managers and physicians over costs dissolves when the qualitative context and the significant variance in quantitative scores (see Figure 3) are taken into account.

For Cost-Focused managers, budget constraints are immediate daily pressures: payroll commitments, IT system licensing fees (*one respondent cited ~100,000 € annually for existing software maintenance alone*), and the inability to generate the 5% co-financing required for EU structural grants. For physicians, cost is an abstract frustration; they are aware of financial limitations but do not confront them operationally. Neither group is philosophically opposed to AI; they are operating from different information environments regarding institutional finances. Several respondents in financial roles also noted that “budget lack” sometimes functions as a rhetorical device, a socially acceptable explanation for resistance whose actual roots lie in uncertainty and fear. The cost-divergence finding among senior managers parallels the operational-financial sensitivity observed in adjacent healthcare implementation studies, including the segmentation work of Koziel and Shen (32).

Sector equivalence explained. The null finding across public and private sectors (all Mann–Whitney *p* > 0.05) was consistently attributed in the IDIs to workforce circulation and the blurring of boundaries between ownership form and funding source, as many private entities operate primarily on public (NFZ) contracts. The Polish healthcare labor market is characterized by extensive dual employment: physicians and managers frequently hold positions in both public and private institutions simultaneously. They share identical competency deficits, institutional anxieties, and patterns of resistance across sectoral boundaries. Additionally, the private sector’s short-term profit orientation combined with the pressure of high patient expectations (the “paying-demanding” dynamic) paradoxically increases risk aversion for experimental technologies with unclear ROI. As one respondent observed, private entities “*value every penny more*” because their survival depends on profitability, making them less, not more, willing to absorb the financial risk of unproven AI implementations. This mechanism overturns the common assumption that the availability of capital determines technology adoption readiness or that private ownership inherently correlates with higher innovation or perceived service quality.

**Figure 4:**
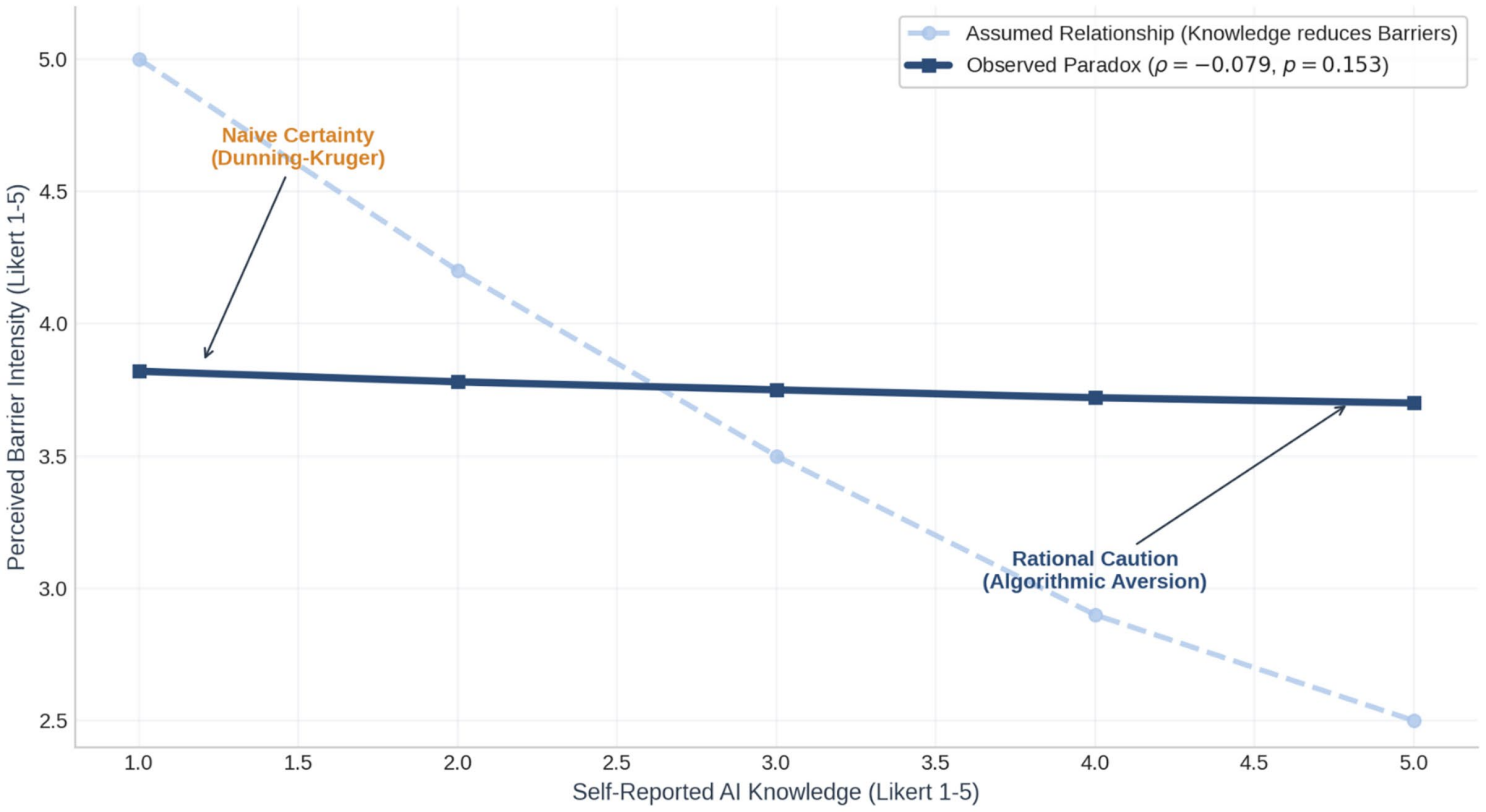
The knowledge paradox: expectation vs. reality. A conceptual visualization of the observed null correlation. The chart illustrates how inflated self-assessment at low knowledge levels and rational caution at high knowledge levels converge, resulting in a flat barrier-perception profile. This chart represents a theoretical heuristic model synthesized from qualitative narratives (Phase 2), rather than plotted quantitative data points.

Knowledge paradox explained. The null correlation between self-reported AI knowledge and barrier perception (*ρ* = −0.079, *p* = 0.153) was explained by two complementary qualitative mechanisms (heuristically modeled in Figure 4).

First, the qualitative data suggest that for respondents with genuine technical familiarity, greater awareness of algorithmic error rates, probabilistic limitations, and legal liability implications offsets the expected reduction in barrier perception as competence grows. This critical caution explains why the negative correlation remains weak (*ρ* = −0.079) and statistically insignificant, rather than indicating a strong barrier-reducing effect. Respondents with actual AI expertise described conducting formal risk assessments that tempered initial enthusiasm, a rational response that may offset the barrier-reducing effect of competence at the population level, which aligns with the phenomenon of algorithmic aversion in high-stakes environments (33, 34). Second, the null correlation partly reflects a measurement artifact consistent with the Dunning-Kruger effect (35, 36): inflated self-assessment means that many “high knowledge” respondents are laypeople by any objective measure. While some literature suggests this effect may be partially a statistical artifact resulting from regression to the mean (37), recent reevaluations confirm that its psychological core remains empirically robust (38). As recently demonstrated in the context of technology adoption, individuals with superficial AI knowledge often overestimate their actual competence (39), using popular-science terminology that masks superficial understanding. This has direct implications for training design: general awareness campaigns may heighten anxiety by revealing risks without providing the professional-grade competency needed to manage them.

#### Qualitative validation of psychographic clusters

This subsection assesses how closely the four data-derived psychographic clusters map onto the thematic patterns observed in the senior-manager interviews.

The IDI thematic patterns confirm the behavioral reality of the k-means clusters, providing evidence that these are genuine behavioral archetypes rather than merely statistical artifacts. The Skeptic cluster’s blanket resistance, all barrier scores exceeding 4.2, traces to institutional exhaustion documented extensively in the Block 2 IDI material (Block 2 of the interview protocol, which addressed perceived barriers and resistance mechanisms; see Methods). Their resistance is not irrational; it is a pragmatic response to decades of underfunded reform mandates that consumed resources without delivering promised improvements.

Respondents whose profiles mapped to the Skeptic cluster characteristically described AI resistance not as a reasoned response to a specific technology but as a generalized institutional posture, a learned refusal extended uniformly to any incoming change. A representative of a national managers’ association put this in deliberately blunt terms:

*“They are sceptical about everything. Whatever the National Fund introduces, whatever new requirement comes in, they see it as just more work being piled on, as interference with their job. Better to leave things as they were. They complain that there is no money, that everything is pointless, but at the same time they have grown used to administering.” [General_Management-National_Association]*

The mechanism behind this posture, as several senior managers articulated, is the displacement of strategic capacity by chronic operational firefighting. The same respondent diagnosed the condition directly:

*“Directors are not managing. There is no strategic management; there is administration — putting out fires. And it comes from the top down. The founding bodies behave the same way. There is very little strategy in any of this. Directors fight every day just to survive: whether the National Fund will pay, whether there will be money for salaries, whether they can hold on to their doctors.” [General_Management-National_Association]*

This finding aligns directly with the concept of change fatigue, well-documented in recent literature (40, 41). These studies demonstrate that repetitive organizational restructurings and continuous digitalization mandates deplete employees’ psychological resources, resulting in systemic exhaustion and a generalized, self-protective resistance against any subsequent technological innovation.

The Cost-Focused cluster’s selective pragmatism, characterized by the highest cost scores but moderate culture scores, was confirmed in Block 3 fiscal discourse. These respondents focused on immediate operational constraints (payroll, infrastructure) while expressing relatively low concern about organizational culture, consistent with their quantitative profile. They are pragmatists who would engage with AI if a clear financial case were presented.

The Pragmatist cluster’s clinical liability anchoring - second-highest expertise and culture scores, but moderate costs - was confirmed by Block 4 material (Block 4 of the interview protocol, which addressed legal-regulatory and educational requirements). These respondents, predominantly physicians and medical directors, framed resistance through the lens of professional responsibility and legal accountability. Their concern is not with technology in principle, but with the specific intersection of algorithmic uncertainty and clinical liability. In contrast to the Skeptic cluster’s generalized refusal, respondents whose profiles aligned with the Pragmatist cluster reasoned about AI adoption in explicit cost–benefit terms, framing receptivity as conditional on operational fit rather than as an attitudinal stance. A general director of a small county hospital reasoned through the trade-off in concrete operational language:

*“It depends on the condition of the facility. If a facility is in good shape and can afford the experiment, and sees a bigger carrot than the stick, then why not? The simplest place to start is with things that can be counted, for example, automated monitoring of patients' basic vital signs. Then we already have something countable, and we can convert it into money.” [General_Management-Small_County_Hospital]*

The Optimists’ consistently low (moderate in absolute terms) barrier scores across all dimensions were confirmed through expressions of frustration with analog legacy systems and generational resistance rather than with the technology itself. This group views organizational inertia, not AI, as the primary barrier to healthcare improvement.

This multi-method validation approach aligns with Creswell’s (18) framework for cluster validation through qualitative methods, in which statistical typologies are assessed against lived experience data to determine whether mathematical groupings correspond to recognizable behavioral patterns. The present findings confirm this correspondence across all four clusters.

#### Meta-inferences from integration

This subsection presents the three meta-inferences that emerge only when the quantitative and qualitative phases are read against each other.

The systematic integration of survey data with in-depth interview analysis produced meta-inferences inaccessible to either method alone. Two meta-inferences warrant explicit articulation to satisfy the GRAMMS criterion (20). First, what appears as “resistance” in quantitative barrier scores is, in qualitative reality, a spectrum ranging from fiscal pragmatism (Cost-Focused managers) to psychological defense mechanisms (Skeptics), requiring fundamentally different interventions for groups whose survey data codes have similar numeric values. A manager scoring 4.2 on cost barriers because she cannot meet payroll requires a different intervention than a manager scoring 4.2 on cost barriers because he uses “budget lack” as a socially acceptable deflection of deeper fears. Quantitative data alone cannot distinguish these cases; qualitative integration is required. Second, the closing question of the IDIs asking respondents to identify a single systemic change required for AI implementation produced no dominant consensus (no response category reached the ≥7 sources threshold). This absence of consensus is itself an analytically significant finding. It reveals that the interdependence between Outer Setting barriers (legal/regulatory) and Individuals domain barriers (expertise/skills) under the CFIR framework is empirically confirmed. The integrated reading of the two phases produces a third meta-inference: legal-regulatory requirements and educational/competency development function as complementary, mutually reinforcing constraints rather than competing demands. A deputy director responsible for financial and administrative affairs grounded this in the institutional reality:

*“For medicine, the issue is legal - responsibility for the outcome. The regulations governing how work is organized are fifteen or even several dozen years old. They do not keep pace with current developments. This is not a question of money. It is a bureaucratic and legal barrier from our decision-makers, the absence of orders from the President of the National Health Fund and the absence of regulations from the Ministry of Health. This is a very significant barrier.” [Operational_Financial-Large_City_Hospital]*

The regulatory dimension must be reframed as ‘regulatory-supervisory’, extending beyond legislation to include formal certification bodies and hard assessment protocols. The educational dimension must be reframed as ‘leadership and change management’ - extending beyond technical training to include communication strategy, KPI-based benefit demonstration, and generational bridge-building. The distinction is accessible only through the integrated reading of both phases. These reframing emerged only through the integration of the quantitative barrier structure (confirming that barriers mapped to different CFIR domains operate independently) with qualitative organizational narratives.

### Practical implications

Based on the convergent findings from both study phases, the following implementation recommendations are proposed:

1. Target psychographic personas, not job titles. Implementation strategies designed for “physicians” or “managers” as homogeneous groups will underperform strategies that address the specific motivational profiles of Skeptics, Cost-Focused professionals, Pragmatists, and Optimists.
2. Address the Skeptic cluster through trust-building and pilot demonstration. Generic technology promotion increases resistance in this group. Small-scale, reversible pilot implementations with transparent outcome reporting are required to overcome institutional distrust rooted in prior reform failures.
3. Address the Pragmatist cluster through liability-specific protocol training. Clinician resistance is rational and legally anchored. Training must address algorithm override criteria, liability allocation frameworks, and the specific regulatory status of AI-assisted clinical decisions, not general AI awareness.
4. Develop universal competency programs. The cross-role consensus on expertise barriers (*p* = 0.243) indicates that competency development must target all professional groups, not physicians alone.
5. Disaggregate “cost barriers” analytically. The wide variance in cost perception (SD = 1.01, highest among all barriers) reflects genuinely heterogeneous financial realities across facility types. Interventions must distinguish between large clinical hospitals with discretionary research budgets and small county hospitals operating at subsistence levels.
6. Abandon sector-based implementation strategies. The null finding across public and private sectors, explained by workforce homogenization, indicates that sector-specific approaches are misguided. Implementation programs should be designed for cross-sectoral application.
7. Redesign the AI knowledge assessment instrument. The inflated self-assessment phenomenon identified in the IDIs undermines the validity of self-reported knowledge measures. Future research and training needs assessments should employ validated AI literacy instruments with objective performance components.
8. Prioritize ROI articulation. IDI data confirm that, in the absence of a clear business case, even well-funded organizations freeze AI investments. Implementation teams should develop sector-specific ROI models, distinguishing between quality-driven ROI (relevant for clinical hospitals) and efficiency-driven ROI (relevant for private entities) before technical deployment.

### Comparison to prior literature

The present findings converge with and extend several recent contributions to the AI implementation literature. Henzler et al. (1) identified psychological and organizational factors as primary barriers to AI acceptance among healthcare professionals in a systematic review of 2017–2024 studies. Yousif et al. (11) found that healthcare providers’ lack of knowledge and trust were the main barriers to AI implementation across multiple countries, consistent with our competency consensus finding, though without the psychographic segmentation that reveals heterogeneity beneath that consensus.

Our findings extend recent CFIR-based analyses of healthcare AI implementation. Finkelstein et al. (8) applied CFIR to AI-assisted clinical decision support, identifying Inner Setting factors (organizational readiness, available resources) as primary determinants consistent with our finding that organizational culture and infrastructure barriers rank among the top concerns. Peek et al. (9) used CFIR to bridge the gap between AI development and clinical deployment, noting that individual-level knowledge deficits interact with outer-setting regulatory uncertainty to create compound resistance. Swart et al. (10) demonstrated the utility of implementation science frameworks for AI deployment in clinical settings, further supporting the multi-level approach adopted here. Our psychographic segmentation adds granularity to this picture: what CFIR conceptualizes as ‘Individuals domain barriers’ decomposes into at least four distinct attitudinal profiles (Skeptics, Cost-Focused, Pragmatists, Optimists), each requiring differentiated intervention strategies. This granular approach extends current understanding of algorithmic aversion, demonstrating that such resistance is heavily dependent on clinical risk perception (33, 34). The integration logic adopted here - under which qualitative findings explain quantitative null patterns - follows the approach recommended for healthcare implementation research by Arends et al. (19). Within the specific Polish and Central European context, Kowalewska (15) conducted a mixed-methods study of physician AI adoption in Poland and found that physicians’ attitudes were shaped by practical experience and perceived reliability rather than by demographic variables - a finding consistent with our Pragmatist cluster profile. Smółka and Smółka (13) documented Poland’s position on digital health indices relative to EU peers, identifying infrastructure gaps and workforce readiness as systemic challenges. Emerging evidence from Polish primary care settings further corroborates the patient-provider interface (42). The present study extends these findings by demonstrating that these challenges are not uniformly distributed across the workforce but cluster into distinct psychographic profiles requiring differentiated interventions.

#### Limitations

##### Quantitative limitations

The non-probability sampling strategy, while necessary given the absence of a comprehensive national professional registry, limits generalizability. The sample may overrepresent professionals with an existing interest in technology, potentially biasing barrier perception scores downward relative to the full population. K-means clustering results are sensitive to initial centroid placement and the number of clusters specified; although the elbow method was used to determine K = 4, alternative clustering algorithms (e.g., hierarchical clustering) might yield different groupings. The cross-sectional design precludes causal inference regarding the relationship between knowledge, role, and barrier perception. Construct validity of the five barrier dimensions rests on theoretical mapping to the CFIR framework and on internal consistency reliability (Cronbach *α* = 0.844). Independent confirmatory factor analysis and convergent/discriminant validity assessment on a fresh sample are recommended for future work.

##### Qualitative limitations

The purposive sample of *N* = 13 is sufficient for thematic saturation (29) but represents a specific subset of the healthcare workforce: senior management. Front-line clinicians, nursing staff, and IT professionals may hold different perspectives not captured in this analysis. Self-reported narratives are subject to social desirability bias and retrospective recall bias. Participants who volunteered for in-depth interviews may hold more polarized views, either more enthusiastic or more resistant than the survey median, potentially amplifying the extremity of qualitative themes relative to population-level attitudes. The deliberate restriction of the qualitative informant pool to senior managers limits the transferability of the mechanism-level findings to other professional groups. Subsequent qualitative work targeting frontline clinicians and nursing staff could extend or qualify the mechanisms identified here, particularly where Phase 1 detected non-significant trends that may nonetheless harbor sub-group heterogeneity below the present study’s statistical power.

##### Mixed-methods integration limitations

The “connecting” strategy used thematic criteria rather than confirmed cluster assignment for IDI participant selection. Cluster membership could not be verified at the recruitment stage because k-means assignment requires survey completion; cluster-IDI matching was performed *post hoc* through attitudinal alignment analysis. This introduces inferential uncertainty in the Joint Display the cluster assignments of individual IDI respondents are plausible rather than confirmed. The temporal gap between survey data collection (April–September 2025) and IDI recruitment may capture shifting perceptions in a rapidly evolving technology landscape, though no major policy or regulatory changes occurred during the interval.

##### Future research directions

Longitudinal designs that track barrier evolution before and after pilot AI implementations would address the causal limitations of the current cross-sectional approach. Validated AI literacy instruments with objective performance components should replace self-reported knowledge measures to resolve the inflated self-assessment artifact. Replication in other post-socialist healthcare systems would establish whether the psychographic profiles identified here generalize to structurally similar contexts or reflect Poland-specific institutional dynamics.

## Conclusion

This study provides the first mixed-methods investigation encompassing all major professional groups (physicians, managers, allied professionals) in Polish healthcare, demonstrating that professional role has minimal predictive power for perceptions of barriers. Four psychographic personas Skeptics, Cost-Focused, Pragmatists, and Optimists, represent the actual structure of implementation resistance, cross-cutting traditional occupational categories and requiring differentiated intervention strategies.

The consensus on competency constitutes the study’s primary quantitative finding: all professional groups agree that the workforce is unprepared for AI, transcending demographic categories. The qualitative phase revealed that this agreement is amplified by and masks heterogeneous mechanisms: “lack of expertise” functions not only as a real skills gap but also as a social mask for deeper psycho-organizational anxieties rooted in institutional history, generational dynamics, and legal liability concerns. For Skeptics, it reflects systemic exhaustion from repeated underfunded reform mandates. For Pragmatists, it reflects rational clinical liability calculations. For the Cost-Focused, it reflects immediate fiscal constraints. For Optimists, it reflects frustration with organizational inertia rather than personal inadequacy.

The integration of both methodological strands produced meta-inferences inaccessible to either method alone: what appears as uniform “resistance” in survey data is, in experiential reality, a spectrum from fiscal pragmatism to psychological defense, requiring fundamentally different interventions for groups that quantitative instruments code identically. The interaction between legal and educational solutions, implicit in the distinction between CFIR’s Outer Setting and Individuals domains, is validated by qualitative narratives that reveal them as complementary requirements serving different cluster segments.

Policy implications are direct: implementation strategies must address legal-regulatory, competency, and financial barriers concurrently. The five barrier dimensions, mapping onto distinct CFIR domains (Individuals, Inner Setting, Outer Setting), demonstrated differential patterns across clusters, confirming that these barriers are structurally independent constructs. Addressing one dimension does not automatically reduce the others; rather, it requires concurrent rather than sequential implementation strategies. Each barrier dimension operates through distinct mechanisms for different psychographic groups.

Poland’s healthcare system, with its post-socialist institutional legacies, aging workforce, and dual public-private structure, offers a microcosm of implementation challenges common to professionally stratified, resource-constrained healthcare systems undergoing rapid technological transformation. These findings support the CFIR framework’s multi-level model of implementation determinants and extend it by demonstrating that, within the Individuals domain, attitudinal heterogeneity is better captured by psychographic clustering than by professional role categories.

## Data Availability

The raw data supporting the conclusions of this article will be made available by the authors upon reasonable request, without undue reservation.

## References

[1] Henzler D Schmidt S Koçar A Herdegen S Lindinger GL Maris MT . Healthcare professionals’ perspectives on artificial intelligence in patient care: a systematic review of hindering and facilitating factors on different levels. BMC Health Serv Res. (2025) 25:633. doi: 10.1186/s12913-025-12664-2, 40312413 PMC12046968

[2] Topol EJ. High-performance medicine: the convergence of human and artificial intelligence. Nat Med. (2019) 25:44–56. doi: 10.1038/s41591-018-0300-7, 30617339

[3] Rajpurkar P Chen E Banerjee O Topol EJ. AI in health and medicine. Nat Med. (2022) 28:31–8. doi: 10.1038/s41591-021-01614-0, 35058619

[4] Davenport T Kalakota R. The potential for artificial intelligence in healthcare. Future Healthc J. (2019) 6:94–8. doi: 10.7861/futurehosp.6-2-94, 31363513 PMC6616181

[5] European Parliament and Council of the European Union, “Regulation (EU) 2024/1689 laying down harmonised rules on artificial intelligence (Artificial Intelligence Act).” Off J Eur Union (2024). Available online at: https://eur-lex.europa.eu/eli/reg/2024/1689/oj/eng (Accessed October 26, 2026).

[6] Damschroder LJ Aron DC Keith RE Kirsh SR Alexander JA Lowery JC. Fostering implementation of health services research findings into practice: a consolidated framework for advancing implementation science. Implement Sci. (2009) 4:50. doi: 10.1186/1748-5908-4-50, 19664226 PMC2736161

[7] Damschroder LJ Reardon CM Widerquist MAO Lowery J. The updated consolidated framework for implementation research based on user feedback. Implement Sci. (2022) 17:75. doi: 10.1186/s13012-022-01245-0, 36309746 PMC9617234

[8] Finkelstein J Gabriel A Schmer S Truong T-T Dunn A. Identifying facilitators and barriers to implementation of AI-assisted clinical decision support in an electronic health record system. J Med Syst. (2024) 48:89. doi: 10.1007/s10916-024-02104-9, 39292314 PMC11410896

[9] Peek N Capurro D Rozova V Van Der Veer SN. Bridging the gap: challenges and strategies for the implementation of artificial intelligence-based clinical decision support systems in clinical practice. Yearb Med Inform. (2024) 33:103–14. doi: 10.1055/s-0044-1800729, 40199296 PMC12020628

[10] Swart R Boersma L Fijten R Van Elmpt W Cremers P Jacobs MJG. Implementation strategy for artificial intelligence in radiotherapy: can implementation science help? JCO Clin Cancer Inform. (2024) 8:e2400101. doi: 10.1200/CCI.24.00101, 39705640 PMC11670909

[11] Yousif M Asghar S Akbar J Masood I Arshad MR Naeem J . Exploring the perspectives of healthcare professionals regarding artificial intelligence; acceptance and challenges. BMC Health Serv Res. (2024) 24:1200. doi: 10.1186/s12913-024-11667-9, 39379939 PMC11459946

[12] Białczyk A Leśniak G Nadolny F Mrowiec J Otałęga A. Exploring digital health horizons: a narrative review of e-health innovations in Poland, Spain, Romania and Estonia. Prospects Pharm Sci. (2024) 22:32–7. doi: 10.56782/pps.178

[13] Smółka J Smółka M. "Digital health index in Poland". In: Digitalization and Innovation in Health, 1st Edn. London: Routledge (2024). p. 63–84.

[14] Powzun-Palczuk E Walkowiak D. Ranking and clustering G20 healthcare systems: a framework for U.S. reform. Front Public Health. (2026) 14:1810123. doi: 10.3389/fpubh.2026.1810123, 42040123 PMC13106346

[15] Kowalewska E. Physicians and AI in healthcare: insights from a mixed-methods study in Poland on adoption and challenges. Front Digit Health. (2025) 7:1556921. doi: 10.3389/fdgth.2025.1556921, 40161560 PMC11949901

[16] Castonguay A Wagner G Motulsky A Paré G. AI maturity in health care: an overview of 10 OECD countries. Health Policy. (2024) 140:104938. doi: 10.1016/j.healthpol.2023.104938, 38157771

[17] Blumenthal D Bennett KJ McDonough JE. Overcoming the digital divide in health care AI. N Engl J Med. (2026) 394:1876–7. doi: 10.1056/NEJMp2600816, 42112869

[18] Creswell JW Creswell JD. Research design: Qualitative, Quantitative, and Mixed Methods Approaches. 6th ed. Los Angeles London New Delhi Singapore Washington DC Melbourne: Sage (2023).

[19] Arends BKO McCormick JM Van Der Harst P Heus P Van Es R. Barriers, facilitators and strategies for the implementation of artificial intelligence-based electrocardiogram interpretation: a mixed-methods study. Eur J Clin Investig. (2025) 55:e14387. doi: 10.1111/eci.14387

[20] O’cathain A Murphy E Nicholl J. The quality of mixed methods studies in health services research. J Health Serv Res Policy. (2008) 13:92–8. doi: 10.1258/jhsrp.2007.007074, 18416914

[21] Brancato G. Macchia S. Murgia M. Signore M. Simeoni G. Blanke K. . “Handbook of Recommended Practices for Questionnaire Development and Testing in the European Statistical System.” European Commission Grant Agreement 200410300002 (2006) (Accessed October 13, 2025). Available online at: https://www.istat.it/it/files/2013/12/Handbook_questionnaire_development_2006.pdf

[22] Cronbach LJ. Coefficient alpha and the internal structure of tests. Psychometrika. (1951) 16:297–334. doi: 10.1007/BF02310555

[23] DeVellis RF. "Scale development: theory and applications". In: Applied Social Research Methods Series, vol. 26, 4th Edn. Los Angeles, CA; London; New Delhi; Singapore; Washington, DC; Melbourne: SAGE (2017)

[24] Nunnally JC Bernstein IH. "Psychometric theory". In: McGraw-Hill Series in Psychology, 3rd Edn. New York: McGraw-Hill (1994)

[25] Conover W. Iman R. (1979). Multiple-comparisons procedures. Informal report: LA-7677-MS, 6057803.

[26] Dunn OJ. Multiple comparisons using rank sums. Technometrics. (1964) 6:241–52. doi: 10.1080/00401706.1964.10490181

[27] Braun V Clarke V. Reflecting on reflexive thematic analysis. Qual Res Sport Exerc Health. (2019) 11:589–97. doi: 10.1080/2159676X.2019.1628806

[28] Braun V Clarke V. Thematic Analysis: A Practical Guide. Los Angeles, CA; London; New Delhi; Singapore; Washington, DC; Melbourne: Sage (2022).

[29] Guest G Bunce A Johnson L. How many interviews are enough? An experiment with data saturation and variability. Field Methods. (2006) 18:59–82. doi: 10.1177/1525822X05279903

[30] Fetters MD Curry LA Creswell JW. Achieving integration in mixed methods designs—principles and practices. Health Serv Res. (2013) 48:2134–56. doi: 10.1111/1475-6773.12117, 24279835 PMC4097839

[31] Smith E Ibanez A Lavretsky H Berk M Eyre HA. Psychographic segmentation: another lever for precision population brain health. Front Aging Neurosci. (2021) 13:783297. doi: 10.3389/fnagi.2021.783297, 34955814 PMC8692771

[32] Koziel AM Shen C. Psychographic and demographic segmentation and customer profiling in mobile fintech services. Kybernetes. (2025) 54:1262–88. doi: 10.1108/K-07-2023-1251

[33] Bankuoru Egala S Liang D. Algorithm aversion to mobile clinical decision support among clinicians: a choice-based conjoint analysis. Eur J Inf Syst. (2024) 33:1016–32. doi: 10.1080/0960085X.2023.2251927

[34] Filiz I Judek JR Lorenz M Spiwoks M. The extent of algorithm aversion in decision-making situations with varying gravity. PLoS One. (2023) 18:e0278751. doi: 10.1371/journal.pone.0278751, 36809526 PMC9942970

[35] Kruger J Dunning D. Unskilled and unaware of it: how difficulties in recognizing one’s own incompetence lead to inflated self-assessments. J Pers Soc Psychol. (1999) 77:1121–34. doi: 10.1037/0022-3514.77.6.1121, 10626367

[36] Ehrlinger J Johnson K Banner M Dunning D Kruger J. Why the unskilled are unaware: further explorations of (absent) self-insight among the incompetent. Organ Behav Hum Decis Process. (2008) 105:98–121. doi: 10.1016/j.obhdp.2007.05.002, 19568317 PMC2702783

[37] Gignac GE Zajenkowski M. The Dunning-Kruger effect is (mostly) a statistical artefact: valid approaches to testing the hypothesis with individual differences data. Intelligence. (2020) 80:101449. doi: 10.1016/j.intell.2020.101449

[38] Dunkel CS Nedelec J Van Der Linden D. Reevaluating the Dunning-Kruger effect: a response to and replication of Gignac and Zajenkowski (2020). Intelligence. (2023) 96:101717. doi: 10.1016/j.intell.2022.101717

[39] Guan J He X Su Y Zhang X. The Dunning–Kruger effect and artificial intelligence: knowledge, self-efficacy and acceptance. Manag Decis. (2025) 63:3786–802. doi: 10.1108/MD-06-2023-0893

[40] Beaulieu L Seneviratne C Nowell L. Change fatigue in nursing: an integrative review. J Adv Nurs. (2023) 79:454–70. doi: 10.1111/jan.15546, 36534455

[41] De Vries MSE De Vries MS. Repetitive reorganizations, uncertainty and change fatigue. Public Money Manag. (2023) 43:126–35. doi: 10.1080/09540962.2021.1905258

[42] Kęczkowska J Płaza M Henrykowska G. Expectations and concerns of primary healthcare patients in rural areas and small towns in Poland regarding artificial intelligence. Sci Rep. (2026) 16:7062. doi: 10.1038/s41598-026-37779-2, 41634082 PMC12921287

