# Supplementary material for: The anatomy of AI implementation skepticism in Polish healthcare: an explanatory mixed-methods analysis of psychographic barriers among healthcare professionals
Source: Front Public Health. 2026 Jun 15;14:1866364. doi: 10.3389/fpubh.2026.1866364 (PMC13311006; doi:10.3389/fpubh.2026.1866364)
Supplement: Supplementary file 1 [file Supplementary_file_1.docx]

## Supplementary Materials

**Supplementary Table S1**. Sample descriptive statistics (N=330). All values derived verbatim from the Sample characteristics section of the manuscript.

| **Variable** | **Category** | **n** | **%** |
| --- | --- | --- | --- |
| Professional role | Managers and administrative staff | 156 | 47.3 |
|  | Physicians | 77 | 23.3 |
|  | Allied medical professionals | 97 | 29.4 |
| Sector | Public facilities | 195 | 59.1 |
|  | Private facilities | 101 | 30.6 |
|  | Non-clinical healthcare entities | 34 | 10.3 |
| Sex | Female | 212 | 64.2 |
|  | Male | 116 | 35.2 |
|  | Not specified / Other | 2 | 0.6 |
| Age | Median age category | 51-60 years | |
| Professional experience | >20 years | 157 | 47.6 |
|  | ≤20 years | 173 | 52.4 |
| AI engagement | Currently using AI tools | 150 | 45.5 |
|  | Open to future use | 145 | 43.9 |
|  | No plans to engage with AI | 26 | 7.9 |
| Self-reported AI knowledge | Mean (5-point Likert scale) | 2.98 | |

*Notes: All percentages computed from N=330. AI engagement categories sum to 97.3%; remaining 2.7% (n≈9) classified as "unsure/other" in the original survey response. Counts for sex and experience categories computed by applying reported percentages to N=330 and rounding to the nearest integer.*

**Supplementary Table S2**. Median and IQR for each barrier dimension stratified by professional role (N=330).

| **Barrier dimension** | **Managers & administration staff (n=156)** | | **Physicians (n=77)** | | **Other medical professions (n=97)** | | **Kruskal- Wallis H** | **p** |
| --- | --- | --- | --- | --- | --- | --- | --- | --- |
|  | Med | IQR | Med | IQR | Med | IQR |  |  |
| Lack of expertise & skilled personnel | 4.17 | 3.67 - 4.67 | 4.00 | 3.33 - 4.67 | 4.00 | 3.67 - 4.67 | 2.83 | 0.243 |
| Legal, ethical & regulatory challenges | 4.00 | 3.40 - 4.60 | 3.80 | 3.20 - 4.60 | 3.80 | 3.00 - 4.60 | 3.66 | 0.160 |
| Technical infrastructure & data security | 4.00 | 3.40 - 4.60 | 3.80 | 3.40 - 4.40 | 3.80 | 3.20 - 4.40 | 5.93 | 0.051 |
| Organizational culture & workforce resistance | 3.75 | 3.00 - 4.25 | 3.75 | 3.00 - 4.25 | 3.75 | 3.00 - 4.25 | 0.49 | 0.784 |
| High implementation and maintenance costs | 4.00 | 3.00 - 5.00 | 3.50 | 2.50 - 4.00 | 3.50 | 3.00 - 4.00 | 12.46 | 0.002 |
